# Cigarette Smoke Modulates Vascular Smooth Muscle Phenotype: Implications for Carotid and Cerebrovascular Disease

**DOI:** 10.1371/journal.pone.0071954

**Published:** 2013-08-14

**Authors:** Robert M. Starke, Muhammad S. Ali, Pascal M. Jabbour, Stavropoula I. Tjoumakaris, Fernando Gonzalez, David M. Hasan, Robert H. Rosenwasser, Gary K. Owens, Walter J. Koch, Aaron S. Dumont

**Affiliations:** 1 Joseph and Marie Field Cerebrovascular Research Laboratory, Division of Neurovascular and Endovascular Surgery, Department of Neurological Surgery, University of Virginia, Charlottesville, Virginia, United States of America; 2 Department of Neurological Surgery, University of Virginia, Charlottesville, Virginia, United States of America; 3 Department of Neurosurgery, University of Iowa, Iowa City, Iowa, United States of America; 4 Department of Molecular Physiology and Biophysics, Robert M. Berne Cardiovascular Research Center, Charlottesville, Virginia, United States of America; 5 Center for Translational Medicine and Department of Pharmacology, Temple University, Philadelphia, Pennsylvania, United States of America; University of Milan, Italy

## Abstract

**Background:**

The role of smooth muscle cell (SMC) phenotypic modulation in the cerebral circulation and pathogenesis of stroke has not been determined. Cigarette smoke is a major risk factor for atherosclerosis, but potential mechanisms are unclear, and its role in SMC phenotypic modulation has not been established.

**Methods and Results:**

In cultured cerebral vascular SMCs, exposure to cigarette smoke extract (CSE) resulted in decreased promoter activity and mRNA expression of key SMC contractile genes (SM-α-actin, SM-22α, SM-MHC) and the transcription factor myocardin in a dose-dependent manner. CSE also induced pro-inflammatory/matrix remodeling genes (MCP-1, MMPs, TNF-α, IL-1β, NF-κB). CSE increased expression of KLF4, a known regulator of SMC differentiation, and siKLF4 inhibited CSE induced suppression of SMC contractile genes and myocardin and activation of inflammatory genes. These mechanisms were confirmed *in vivo* following exposure of rat carotid arteries to CSE. Chromatin immune-precipitation assays *in vivo* and *in vitro* demonstrated that CSE promotes epigenetic changes with binding of KLF4 to the promoter regions of myocardin and SMC marker genes and alterations in promoter acetylation and methylation.

**Conclusion:**

CSE exposure results in phenotypic modulation of cerebral SMC through myocardin and KLF4 dependent mechanisms. These results provides a mechanism by which cigarette smoke induces a pro-inflammatory/matrix remodeling phenotype in SMC and an important pathway for cigarette smoke to contribute to atherosclerosis and stroke.

## Introduction

Cigarette smoking is a major cause of premature death worldwide, and the leading preventable source of morbidity and mortality in the United States. [1] Cigarette smoking is a major risk factor for cerebral vascular injury including atherosclerosis, [2], [3], [4], [5], [6] a key process behind carotid and cerebrovascular disease including ischemic stroke, intracerebral hemorrhage, and cerebral aneurysm formation. [7], [8], [9], [10], [11] Although exposure to chemicals in cigarette smoke has consistently been shown to have a significant effect on various pathways of the cerebral immune and inflammatory response, [12], [13], [14], [15], [16], [17], [18], [19], [20], [21], [22] the means by which cigarette smoking may contribute to the pathogenesis of carotid and cerebrovascular disease has not been clearly defined.

Human and animal studies have shown that cigarette smoke produces abnormal endothelial function in nearly every vascular territory. [14], [15], [16], [17], [18], [19] Studies have found that damage may be secondary to induction of pro-inflammatory mediators, upregulation of immune cells, and generation of reactive oxygen species. [14] The underlying mechanisms have yet to be determined and alterations in cerebral vascular smooth muscle cells (SMC) have been largely ignored.

Unlike terminally differentiated cardiac or skeletal muscle cells, vascular SMC retain remarkable plasticity. [23], [24] In response to environmental stimuli, SMC can undergo changes from cells principally concerned with contraction to cells that are primarily involved in inflammation and matrix remodeling. [23] This phenotypic modulation is defined by a decreased expression of key SMC marker contractile proteins (smooth muscle myosin heavy chain (SM-MHC), SM-α-actin and SM-22 α) with increased expression of inflammatory mediators. [23], [25], [26], [27], [28], [29], [30] Phenotypic modulation of SMC is known to be important in the pathogenesis of vascular injury [28] and atherosclerosis [29] both key elements of cerebrovascular disease. [30] Despite the significant pathological effects of cigarette smoke, studies have not assessed its role in vascular SMC phenotypic modulation or mechanisms directly contributing to cerebrovascular disease.

The aims of the present study were to: (1) To evaluate a potential role of CSE in producing phenotypic modulation of cultured cerebral vascular SMC including repression of SMC marker genes and induction of pro-inflammatory, matrix remodeling genes that may play a critical role in the pathogenesis of cerebrovascular disease (2) To determine if CSE produces similar phenotypic modulation of SMC in carotid arteries *in vivo* (3) To assess whether CSE-induced phenotypic modulation of SMC occurs, at least in part, through downregulation of myocardin, a principle transcription factor involved in expression of SMC contractile marker genes (4) To test the hypothesis that CSE-induced phenotypic modulation of SMC is mediated by Kruppel-like transcription factor 4 (KLF4), a key transcription factor regulating SMC differentiation marker gene expression [23], [27], [31] and induction of somatic cells into pluripotent stem cells. [32].

## Materials and Methods

This study was carried out in strict accordance with the recommendations in the Guide for the Care and Use of Laboratory Animals of the National Institutes of Health. The protocol was approved by the Committee on the Ethics of Animal Experiments of the Thomas Jefferson University (Permit Number: 833). All surgery was performed under Isofluorane anesthesia. All efforts were made to minimize suffering. Cerebral blood vessels (circle of Willis) from rats were harvested for primary cell culture and treated with cigarette smoke extract (CSE; Murty Pharmaceutical) for quantitative PCR, Western blot, chromatin immune-precipitation, and assessment following adenovirus promoter transfection (See Supplementary Materials and Methods). Additionally, *in vivo* experiments were carried out following application of pluronic gel (Sigma-Aldrich) containing CSE to the adventitial surface of rat carotid arteries, a validated model of carotid atherosclerosis and cerebrovascular disease. [27], [33], [34].

### Rat Cerebral Vascular Smooth Muscle Cell Culture and CSE Exposure

Cerebral blood vessels (circle of Willis) from 7 week old rats (Sprague-Dawley) were harvested for primary cell culture. Cerebral blood vessels were placed in cold Hank’s Balanced Salt Solution (HBSS). Vessels were washed, connective tissue and arachnoid were removed and were placed in enzyme solution (Collagenase II 1 mg/ml, Soybean Trypsin Inhibitor 1 mg/ml, and Elastase 0.744 U/ml in HBSS) for 8–10 minutes. The adventitial layer was stripped. After an hour, the vessels were washed in media, plated in culture dishes in growth medium containing Dulbeco’s Modified Eagle Medium/F12 media (20% FBS, and 1% anti-anti) in a humidified atmosphere of 95% air and 5% CO_2_. Cells were weaned to 10% FBS after passage 3–5.

Cells were passaged at 70–80% confluence. For experiments, unless noted otherwise, cells were grown to confluence and starved in serum free media containing DMEM/F12, L-ascorbic acid (3.52 mg/ml), apotransferrin (5 µg/ml) and selenium selenite (6.25 ng/ml) in addition to L-glutamine, anti-anti (Invitrogen) for 72 hours. Cells were treated with cigarette smoke extract (Murty Pharmaceutical) dissolved in HEPES buffer for 24 hours for quantitative real time PCR, 72 hours for Western blot, and 2 hours for chromatin immune-precipitation. The CSE dose and timing was based on our preliminary studies and was selected to approximate levels of water-soluble components of cigarette smoke and nicotine present in plasma levels of human smokers. [14], [35].

### Transient Transfection and Luciferase Assay

Cultured rat cerebral vascular SMC at approximately 75% confluence were transfected with reporter plasmid using FuGENE reagent (Roche Diagnostics Corp.) following the manufacturer’s protocol. The promoter luciferase constructs were obtained from the Owens’ laboratory and included: SM α-actin-luc (−2555/+2813 bp), SM MHC-luc (−4220/+11600 bp), and pGL3 basic plasmid (Promega Corporation). Luciferase activity was measured with luciferase assay substrate (Promega) and normalized to total protein content (Coomassie Plus protein Assay reagent, Pierce).

### Quantitative Real-Time RT-PCR

At the time of harvest, cerebral SMC were washed once with phosphate-buffered saline (PBS) and lysed in 350 µl of RNeasy lysis buffer (Qiagen). Total RNA was prepared using manufacturer’s instructions (RNeasy kit; Qiagen). cDNA was prepared using 0.5–1 µg of total RNA using iScript cDNA synthesis kit (Bio-Rad laboratories). iQ SYBR Green (Bio-Rad laboratories) was used to run quantitative real-time polymerase chain reaction (RT-PCR) using a CFX-96 real time system (Bio-Rad laboratories). Results were normalized to 18S rRNA gene expression. Primer sequences are listed in the online supplemental Table 1.

**Table 1:** Real-time PCR Primer Sequences.

	Forward Primer (Rat)	Reverse Primer (Rat)
1	**SM-α-Actin**	
	AGTCGCCATCAGGAACCTCGAG	ATCTTTTCGATGTCGTCCCAGTTG
2	**SM-MHC**	
	CAGTTGGACACTATGTCAGGGAAA	ATGGAGACAAATGCTAATCAGCC
3	**SM-22α**	
	GCATAAGAGGGAGTTCACAGACA	GCCTTCCCTTTCTAACTGATGATC
4	**Myocardin**	
	CGGATTCGAAGCTGTTGTCTT	AAACCAGGCCCCCTTCC
5	**KLF4**	
	CTTTCCTGCCAGACCAGATG	GGTTTCTCGCCTGTGTGAGT
6	**18S**	
	CGGCTACCACATCCAAGGAA	AGCTGGAATTACCGCGGC
7	**MMP3**	
	GCCAATGCTGAAGCTTTGATGTAC	GGGAGGTCCATAGAGGGATTGAAT
8	**IL-1β**	
	TTGTGCAAGTGTCTGAAGCA	TGTCAGCCTCAAAGAACAGG
9	**MCP1**	
	CTCAGCCAGATGCAGTTAATGC	TCTCCAGCCGACTCATTG G
10	**TNF**-α	
	AAAGCATGATCCGAGATGT	AGCAGGAATGAGAAGAGG C
11	**NF-κB (IκB-α)**	
	GCTGAAGAAGGAGCGCTACT	TCGTACTCCTCGTCTTTCATGGA
12	**MMP9**	
	AAGCCTTGGTGTGGCACGAC	TGGAAATACGCAGGGTTTGC

### Western Blot Analysis

Cerebral SMC were washed twice with ice-cold PBS. Cells were lysed with RIPA buffer (Sigma-Aldrich) containing protease inhibitor mini tablet (Roche) and phosphates inhibitor cocktail (Pierce, Thermo Scientific). Cells were scraped on ice, samples collected and centrifuged at 10,000 g for 10 minutes. Proteins were quantified using BCA Protein Assay (Thermo Fisher). Samples were heated for 60s at 95°C. Samples were loaded in 10% Tris-HCl gel (Bio-Rad), separated and transferred to polyvinyl difluoride membrane. Membrane was blocked for an hour and incubated with primary and secondary antibodies in blocking buffer for an hour each at room temperature. Immunodetection was carried out with enhanced chumiluminescence (ECL) detection substrate (Thermo Scientific). The following antibodies were used: SM α-Actin (1A4: Sigma Chemicals), SM-22α (Abcam), SM-MHC (Biomedical Technologies Inc.), KLF4 (Santa Cruz Biotechnology), and GAPDH (Millipore).

### siKLF4 Transfection

Short interfering double stranded RNAs specific to KLF4 (5′-GUACAAUGGUUUAUUCCA-3′ and 5′-CGAUCUACAUUUAUGACCU-3′) and EGFP (5′-GAACGGCAUCAAGGUGAAC-3′) were purchased from MWG-Operon. Cells were plated at 10,000 cell/cm^2^ overnight and transfected using Oligofectamine (Invitrogen) according to manufacturer’s protocol. Transfection was carried out to greater than 95% efficacy.

### Proliferation Assay and TUNEL Assay

Cerebral vascular SMC were plated in 96 well plates and treated with increasing concentrations of CSE for 24 hours. CyQuant NF (Invitrogen) cell proliferation assay kit was used to study proliferation assay. Manufacturer recommended protocol was used to study proliferation assay.

Apoptotic cells were detected using terminal deoxynucleotidyl transferase–mediated dUTP-biotin nick end-labeling (TUNEL) assay according to manufacturer’s protocol (Invitrogen) after incubating cells with CSE for 24 hours.

### Chromatin Immunoprecipitation

Cerebral vascular SMC were fixed with 1% paraformaldehyde in media for 10 minutes at room temperature to cross-link protein-DNA and protein-protein interactions. Cells were harvested using ChIP-IT Express kit (Active Motif) using manufacturer’s protocol. Chromatin was sheared using sonication into fragments between 200–800 bp. Chromatin-protein complexes were immunoprecipitated using the following antibodies: anti-KLF4 (Santa Cruz Biotechnologies), anti-HDAC2 (Santa Cruz Biotechnologies), and anti-Histone 3-Lysine 9-Acetylation (Millipore), anti-Histone 3-Lysine 27-Tri Methylation (Cell Signaling) and anti-Histone 3-Lysine 4-Dimethylation and Salmon sperm DNA was added to magnetic beads. As a negative control, anti-body was excluded from immunoprecipitation reaction. Samples were washed, reverse cross-linked and purified. Purified DNA was quantified using Picogreen reagent (Molecular Probes; Invitrogen). Real-time RT-PCR was performed to amplify CArG-containing regions of SM-α Actin and SM-MHC promoters. Results are presented as percent of whole chromatin. Primer sequences were as follows: SM α-actin 5-agcagaacagaggaatgcagtggaagagac-3. 5-cctcccactcgcctcccaaacaaggagc-3; SMMHC 5.-ctgcgcgggaccatatttagtcagggggag-3, 5-ctgggcgggagacaacccaaaaaggccagg-3. *In vivo* ChIP was performed on carotid vessels after treating them first with F-127 Pluronic gel containing CSE. Vessels were dissected, washed in ice-cold PBS and snap frozen in liquid nitrogen. Vessels were cut into 5 mm pieces while in 1% paraformaldehyde in media and then rotated for a total of 15 minutes. Vessels were homogenized using Ultra-Turrax (Sigma-Aldrich), lysed and washed with ChIP-IT Express kit (Active Motif) using manufacturer’s protocol. Chromatin was sonicated and the remainder of the aforementioned protocol was followed.

### Application of CSE to Rat Carotid Arteries

*In vivo* experiments were carried out following application of pluronic gel (Sigma-Aldrich) containing CSE to the adventitial surface of rat carotid arteries, a validated model of carotid atherosclerosis and cerebrovascular disease. [27], [33], [34] The animal Care and Use Committee at Thomas Jefferson University approved the animal protocol. Anesthesia was induced and maintained using Isofluorane. 100 µl of ice-cold F-127 Pluronic Gel (Sigma-Aldrich) containing CSE at 0.8 mg/ml (n = 6) and vehicle (n = 6) was applied to the adventitial surface of rat carotid arteries for 6–8 hrs for KLF4 and 24–48 hours for differentiation and inflammatory marker genes. Vessels were extracted, homogenized using Ultra-Turrax (Sigma-Aldrich), and total RNA was extracted using RNeasy fibrous mini kit (Qiagen) using manufacturer’s protocol. Results were normalized to 18S rRNA gene expression and compared to non-treated vessels.

### Statistics

All experiments were performed with a minimum of triplicate samples and were conducted in 3 to 6 independent experiments unless otherwise indicated. Comparison of means between two groups was carried out using independent t-test and analysis of means between 3 or more groups was carried out with ANOVA post hoc Bonferroni correction analysis as appropriate. Error bars represent standard error of the mean (SEM). Results were considered statistically significant for p-values <0.05. Asterisks represent the following: *(<0.05), **(<0.01), ***(<0.001) and ****(<0.0001).

## Results

### CSE Exposure Repressed Myocardin and SMC Marker Gene Expression In Cultured Cerebral Vascular SMC

**Figure 1:**
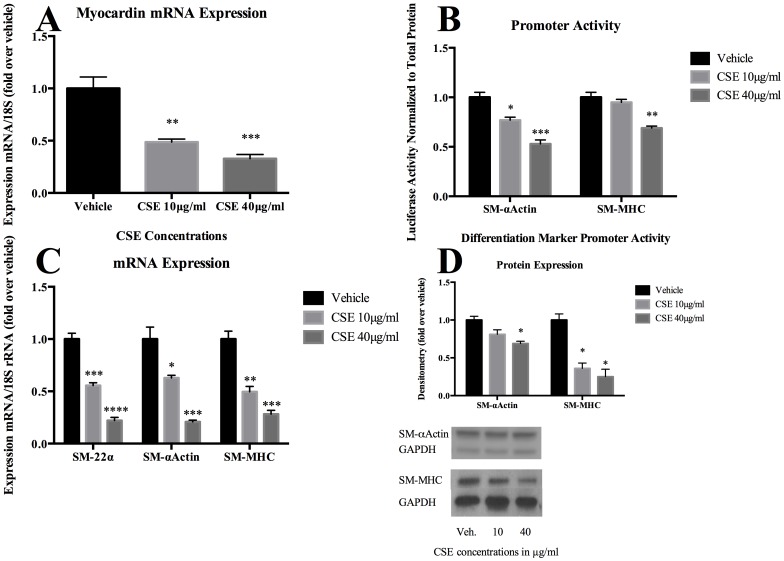
CSE decreases myocardin and SMC marker gene expression. A) Cultured cerebral vascular SMCs were treated with the indicated concentrations of CSE for 24 hours. Myocardin mRNA expression was quantified using real-time RT-PCR and normalized to 18S rRNA. SM MHC-luc and SM α-actin-luc promoter-luciferase constructs were transiently transfected into cerebral vascular SMCs for 24 hours followed by treatment with CSE for 24 hours. Luciferase activity was measured and normalized to total protein content and then expressed as fold increase over vehicle. Values represent mean±SEM. C) cultured cerebral vascular SMCs were treated for 24 hours with the indicated concentration of CSE. Real-time RT-PCR was performed, normalized to 18S rRNA, and expressed as fold increase over vehicle. Values represent mean±SEM. D) Cultured vascular SMCs were starved for 72 hours and further treated with CSE with the indicated range of concentration for another 72 hours. Total protein lysate of SMCs (0.2 µg) were subjected to Western blot analysis of SM-MHC and SM-α-actin protein expression. GAPDH was used as a loading control.

**Figure 2:**
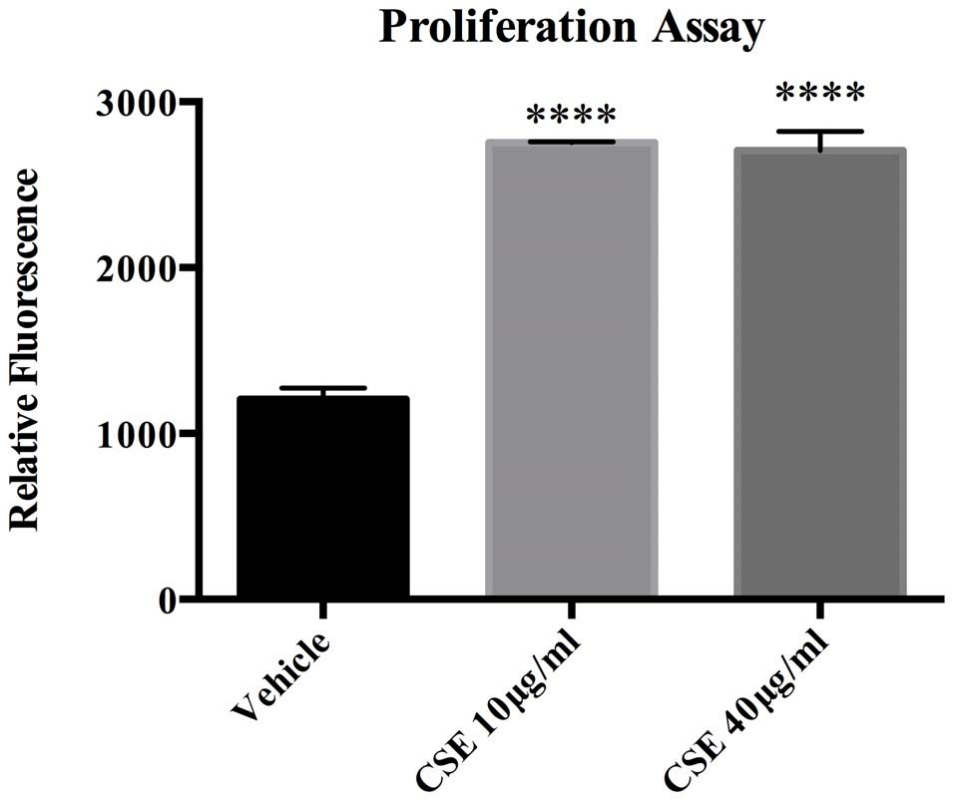
CSE Induced Proliferation. Cerebral VSMC were plated in 96 well plates and treated with increasing concentrations of CSE for 24 hours. CSE significantly increases proliferation in at both 10 µg/ml and 40 µg/ml concentrations.

**Figure 3:**
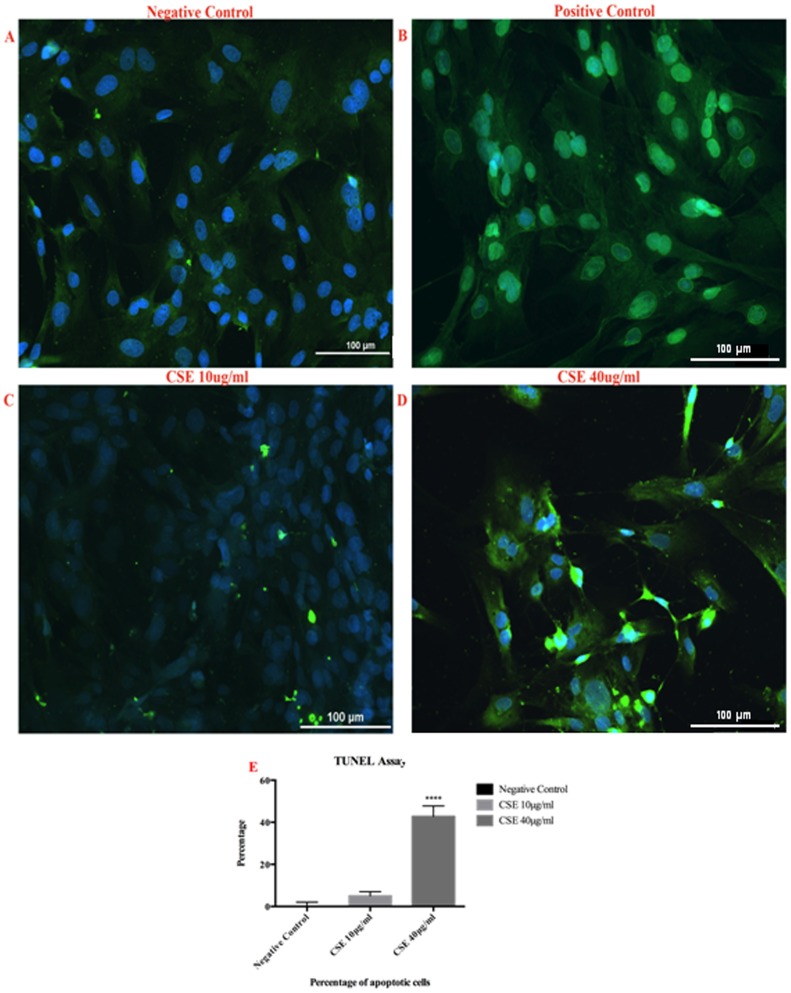
CSE induced dose-dependent apoptosis in SMC. A) Represents negative control. B) Represents positive control. C) Cerebral vascular SMC were incubated with CSE (10 µg/ml) and D) CSE (40 µg/ml) for 24 hours. Click-IT TUNEL assay kit was used to assess for apoptosis. Image J software [64] was used to count apoptotic cells. Data represents percentage of apoptotic cells.

Although a number of factors control vascular SMC differentiation, myocardin has been shown to be a key regulator of SMC differentiation in peripheral vascular beds. [23] To assess the effects of CSE on phenotypic modulation of cerebral vascular SMCs, cultured rat cerebral vascular SMC from the Circle of Willis were treated with CSE for 24 hours. CSE exposure doses were based on preliminary experiments and designed to approximate levels of water-soluble components of cigarette smoke and nicotine present in plasma levels of human smokers. [14], [35] CSE exposure significantly diminished expression of myocardin mRNA in a dose dependent manner (Figure 1A). CSE exposure downregulated SM- α-actin and SM-MHC promoter activity in a dose-dependent manner (Figure 1B). CSE also suppressed SM- α-actin, SM-MHC and SM-22-α mRNA expression in a dose-dependent fashion (Figure 1C). Similarly, SM-α-actin and SM-MHC protein expression decreased following CSE exposure (Figure 1D). Furthermore, CSE induced proliferation (Figure 2), but also initiated apoptosis in vascular SMC in a dose dependent fashion (Figure 3).

### CSE Induced Pro-inflammatory & Matrix Remodeling Phenotypic Modulation of Vascular SMC

**Figure 4:**
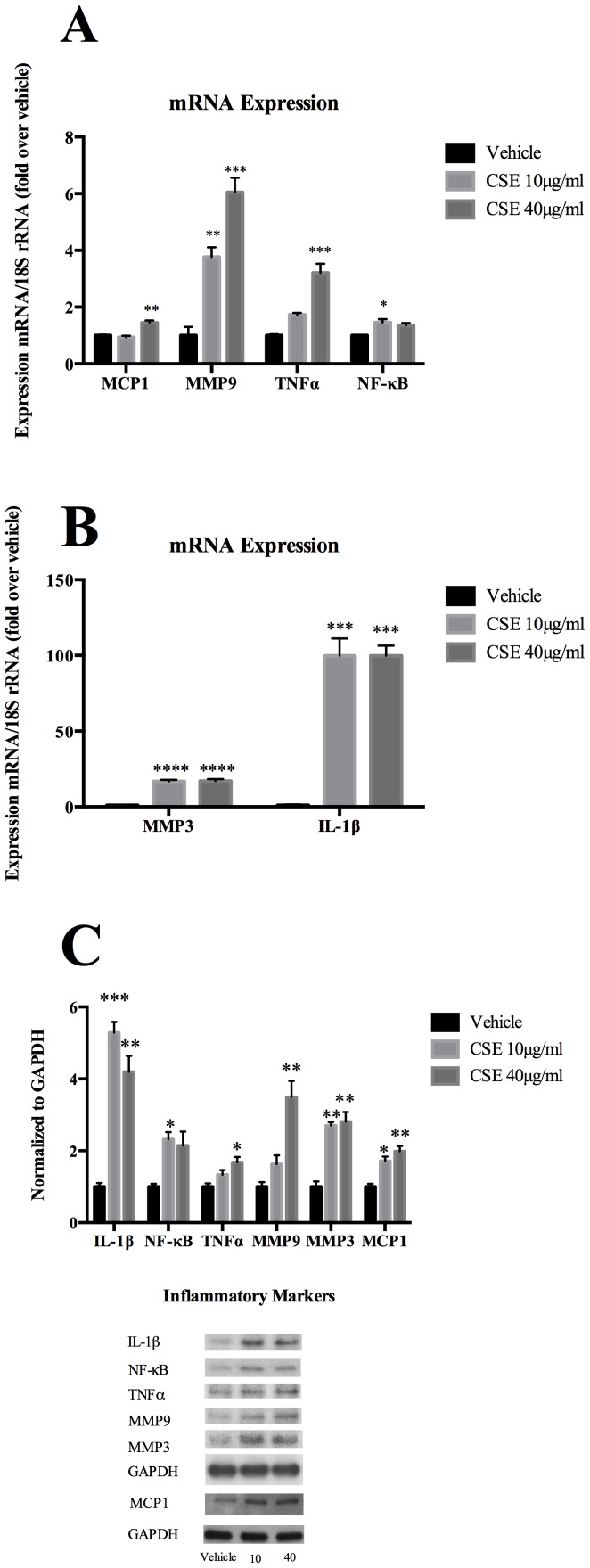
CSE Induced Pro-inflammatory & Matrix Remodeling Phenotypic Modulation. A–B) Cultured cerebral vascular SMCs were treated with the indicated concentrations of CSE for 24 hours. mRNA expression was quantified using real-time RT-PCR and normalized to 18S rRNA. Values represent mean±SEM. C) Cultured cerebral vascular SMCs were starved for 72 hours and further treated with CSE with the indicated range of concentration for another 72 hours. Total protein lysate of SMCs (0.2 µg) was subjected to western blot analysis of MMP-3, MMP-9, TNF-α, IL-1β, NF-κB, and MCP1 protein expression. GAPDH was used as a loading control.

Following various stimuli, SMC can undergo phenotypic modulation and express a number of pro-inflammatory and matrix remodeling genes. [8], [36], [37] Based in part upon established work in the field of peripheral atherosclerosis, [38] experiments were carried out on a number of inflammatory genes that may play a role in carotid and cerebrovascular disease. Quantitative real time PCR and Western blot analysis revealed that CSE exposure for 24 hours markedly increased expression of MCP-1, MMP-3, MMP-9, TNF-α, IL-1β, and NF-κB in a dose-dependent manner (Figure 4).

### Exposure to CSE Induces the Transcription Factor, KLF4, A Key Modulator of SMC Phenotypic Modulation

The transcription factor KLF4 is a key regulator of SMC differentiation. [23], [27], [31] To assess if cerebral vascular SMC differentiation marker gene and pro-inflammatory/matrix remodeling gene expression occurs along with changes in KLF4 expression, cultured cerebral vascular SMC were exposed to CSE. CSE increased early expression of KLF4 as demonstrated in quantitative real time RT-PCR (Online Figure 5A) and occurred in a dose dependent fashion. Similarly, protein expression of KLF4 was increased compared to vehicle (Online Figure 5B).

**Figure 5:**
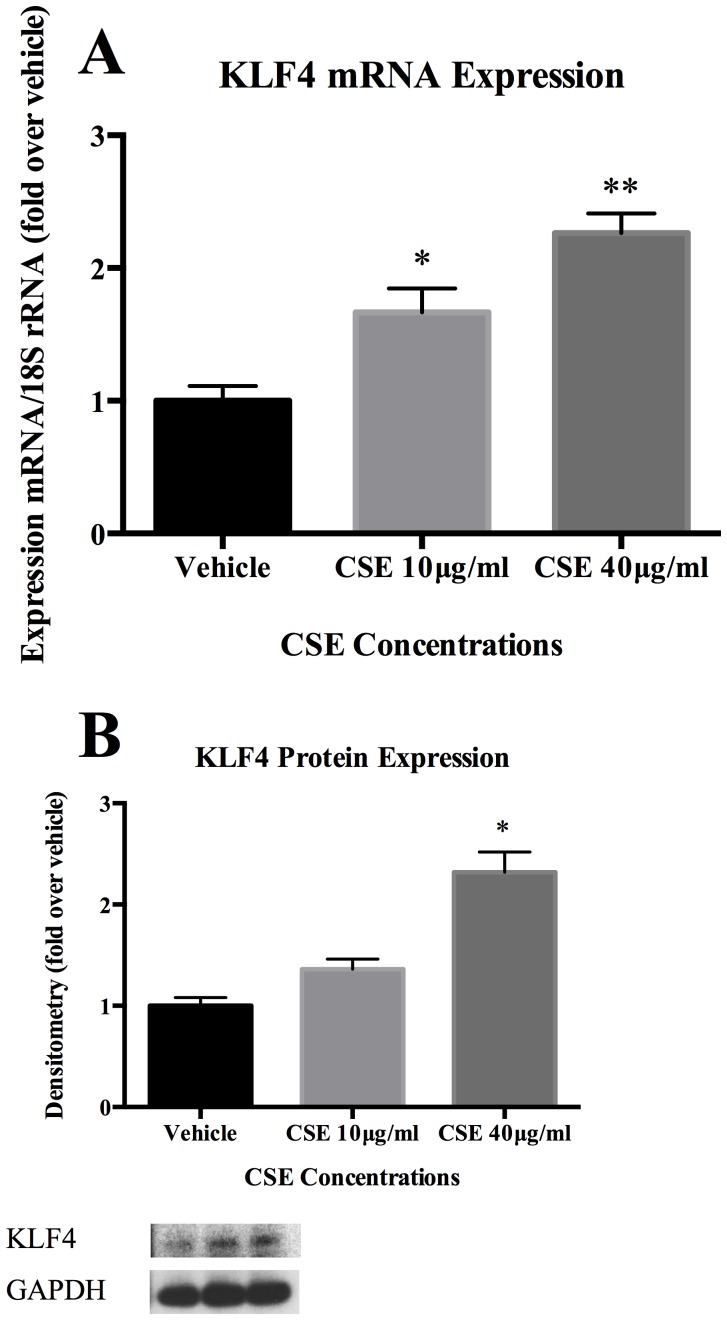
CSE induced expression of the transcription factor, KLF4, a potent regulator of vascular SMC phenotypic modulation. A) SMC were treated for 4 hours with the indicated range of CSE concentrations. Real-time RT-PCR was performed, normalized to 18s rRNA, and expressed as fold increase over vehicle. B) Cultured SMC were starved for 72 hours and further treated with CSE with the indicated range of concentration for another 24 hours. Total protein lysate of SMCs (0.5 µg) were subjected to Western blot analysis of KLF4 protein expression. GAPDH was used as loading control.

### CSE Induced SMC Phenotypic Modulation is Reversed Following KLF4 Inhibition

**Figure 6:**
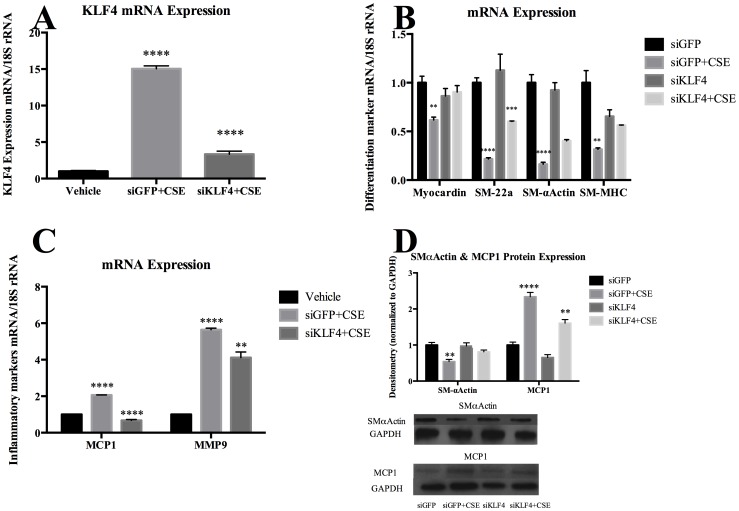
CSE induced suppression of myocardin, SMC marker genes and induction of pro-inflammatory genes was mediated by KLF4. A–C) Cultured cerebral vascular SMCs were transfected with KLF4 siRNA oligonucleotides (siKLF4) or nonspecific control oligonucleotides (siGFP) with CSE (40 µg/ml) or vehicle treatment. Total RNA samples were isolated and myocardin, differentiation marker genes (SM-22α, SM-α-actin and SM-MHC), inflammatory marker genes (MCP1, and MMP9) and KLF4 mRNA expression was analyzed by real-time PCR. Values were normalized to 18S rRNA and represent mean±SEM. The experiment was repeated 4 times and the representative data are shown. D) Three micrograms of total protein lysate of SMC transfected with KLF4 siRNA or GFP siRNA were subjected to western blot analysis using anti-SM-α-actin, anti-MCP1 and anti-GAPDH antibodies. The band intensity was quantified by densitometry. Relative band intensity was normalized to the band intensity of GAPDH and presented as fold-increase over vehicle.

To define whether KLF4 is required for CSE induced phenotypic modulation, cultured cerebral vascular SMC were transfected with siRNA oligonucleotide specific to KLF4 and exposed to CSE. KLF4 siRNA inhibited expression of KLF4 mRNA following exposure to CSE, but GFP control siRNA did not change (Figure 6A). KLF4 siRNA repressed CSE-induced suppression of myocardin as well as SM- α-actin, SM-MHC and SM-22-α while a control GFP siRNA had no effect (Figure 6B). siKLF4 also suppressed CSE-induced expression of inflammatory mRNA expression (Figure 6C) and reversed CSE-induced changes in protein expression (Figure 6D).

### *In Vivo* Exposure of CSE to Rat Carotid Arteries Downregulates Expression of Myocardin and SMC Marker Genes While Upregulating Pro-Inflammatory Genes

**Figure 7:**
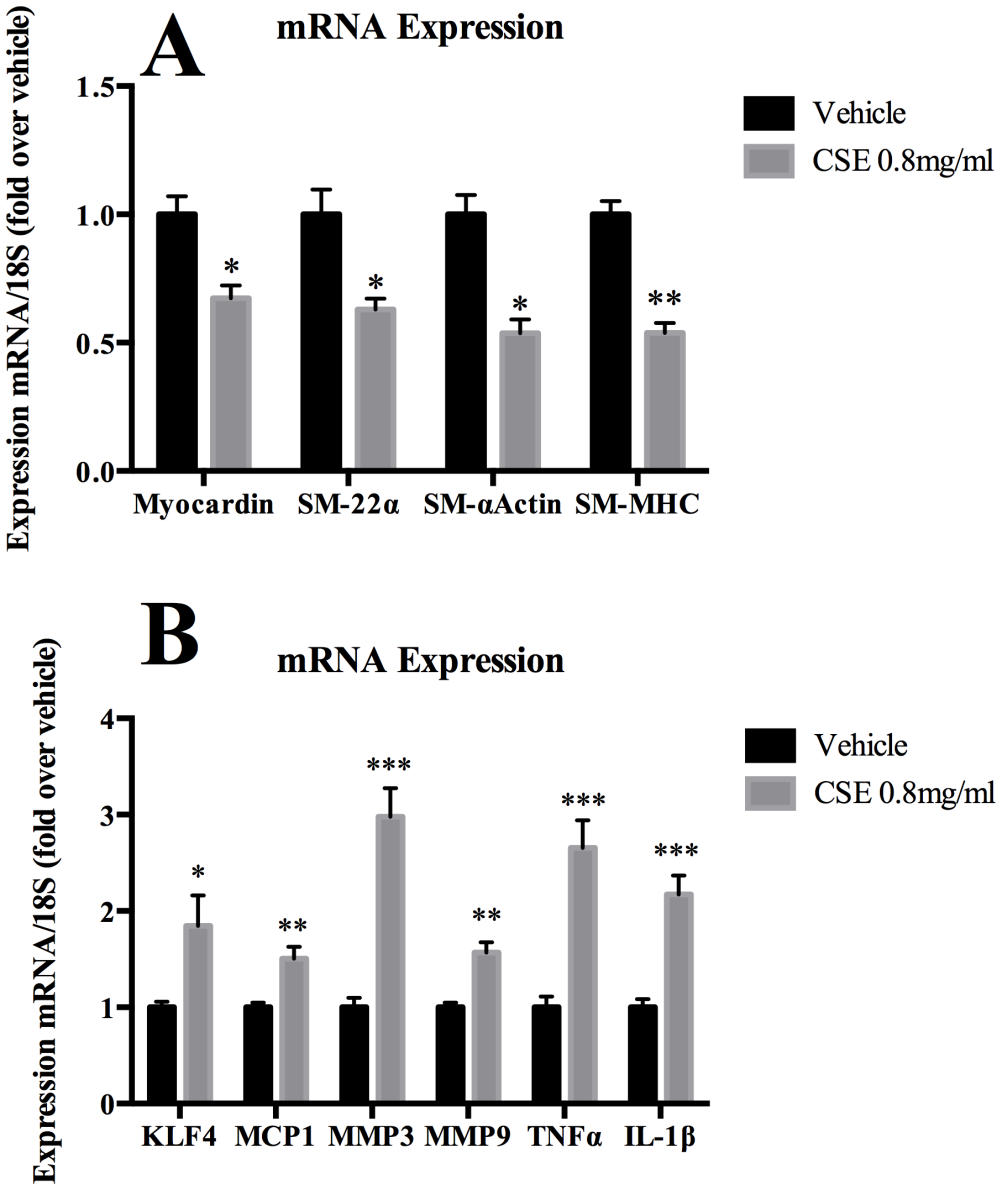
CSE decreased expression of myocardin and SMC marker genes and increased expression of pro-inflammatory genes *in vivo* in rat carotid arteries. Pluronic gel containing CSE (0.8 mg/ml) or vehicle (DMSO) was applied for 24 hours to the adventitial surface of left and right common carotid arteries (n = 6 rats), respectively. Total RNA was isolated from the treated and untreated carotid. A) SMC marker gene and myocardin expression in each sample was normalized to the 18S rRNA level. Values for the left carotid artery were normalized to the right control carotid arteries. B) Similarly, mRNA expression of KLF4 and pro-inflammatory genes was analyzed using real-time RT-PCR.

To define whether CSE produced similar phenotypic modulation *in vivo*, the F-127 pluronic gel system [27], [33], [34] was used to expose the adventitial surface of rat carotid arteries to CSE or vehicle control. Similarly, quantitative real time RT-PCR demonstrated that CSE exposure diminished expression of myocardin as well SM-α-actin, SM-MHC and SM-22-α mRNA compared to control (Figure 7A). CSE exposure also increased KLF4, MCP-1, MMP-3, MMP-9, TNF-α, and IL-1β mRNA expression (Figure 7B). Secondary controls demonstrated that β-actin mRNA was not altered following exposure to CSE and gene expression was not effected in the aorta or liver (data not shown). In summary, these results demonstrate that CSE exposure downregulates expression of myocardin and vascular SMC differentiation genes concerned with contractile function, and upregulates expression of KLF4 and pro-inflammatory/matrix remodeling genes. Furthermore, KLF4 likely suppresses expression of cerebral vascular SMC differentiation genes, in part through KLF4 mediated repression of myocardin.

### CSE Promotes KLF4 Binding to Promoter Regions of Myocardin and Cerebral Vascular SMC Marker Genes Both *In Vitro* and *In Vivo*

**Figure 8:**
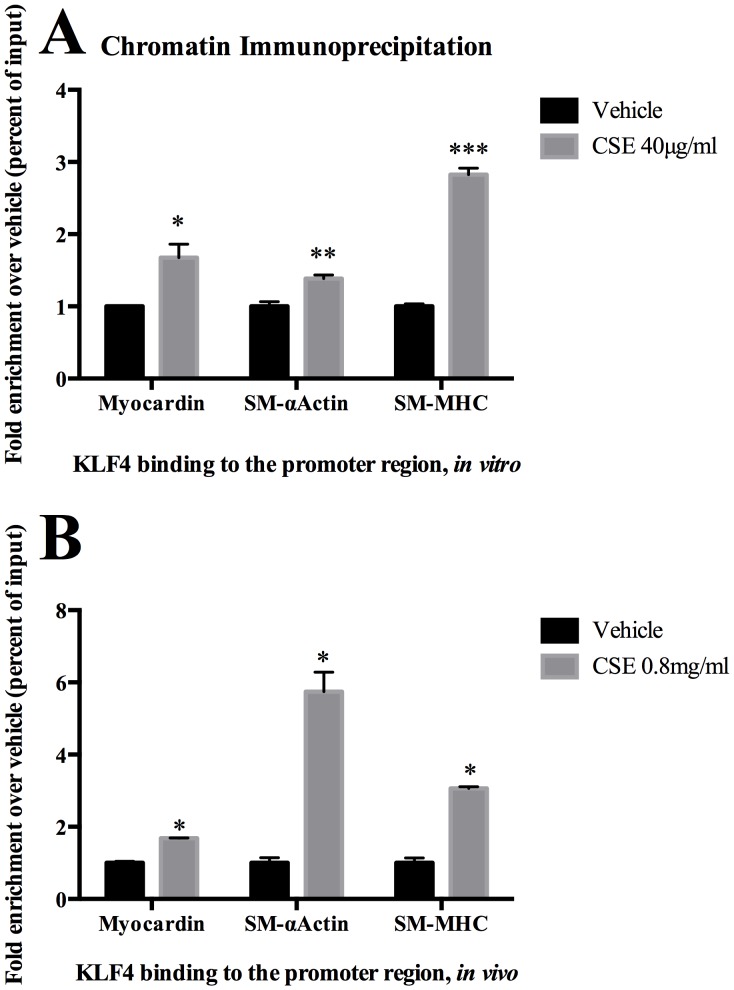
CSE induced binding of KLF4 to the promoter regions of SMC marker genes and myocardin. A) Cultured cerebral vascular SMC were treated with CSE (40 µg/ml) or vehicle (DMSO) for 2 hours. Association of KLF4 to CArG-containing promoter region of SMC marker genes (SM-α-actin and SM-MHC) and myocardin was determined by ChIP assays using anti-KLF4 antibody. Values were normalized to input DNA and compared with vehicle. B) *In vivo* experiments using CSE (0.8 mg/ml) applied as pluronic gel to the adventitial surface of rat (n = 12) carotid arteries for 24 hours. Vessels were harvested and ChIP assays performed as detailed in Methods. Values represent fold-increase over vehicle.

CHIP assays were carried out to define whether there is a direct interaction between KLF4 and the promoter regions of myocardin, and vascular SMC marker genes. Exposure of cerebral SMC to CSE induced KLF4 binding to the promoter region of myocardin, SM- α-actin and SM-MHC (Figure 8A). This was also confirmed *in vivo* through CHIP assays following exposure of rat carotid arteries to pluronic gel containing CSE (Figure 8B).

### Epigenetic Mechanisms Mediating CSE Induced Suppression of Cerebral Vascular SMC Marker Genes Include Recruitment of HDAC2, Histone Hypoacetylation, and Changes in Methylation

**Figure 9:**
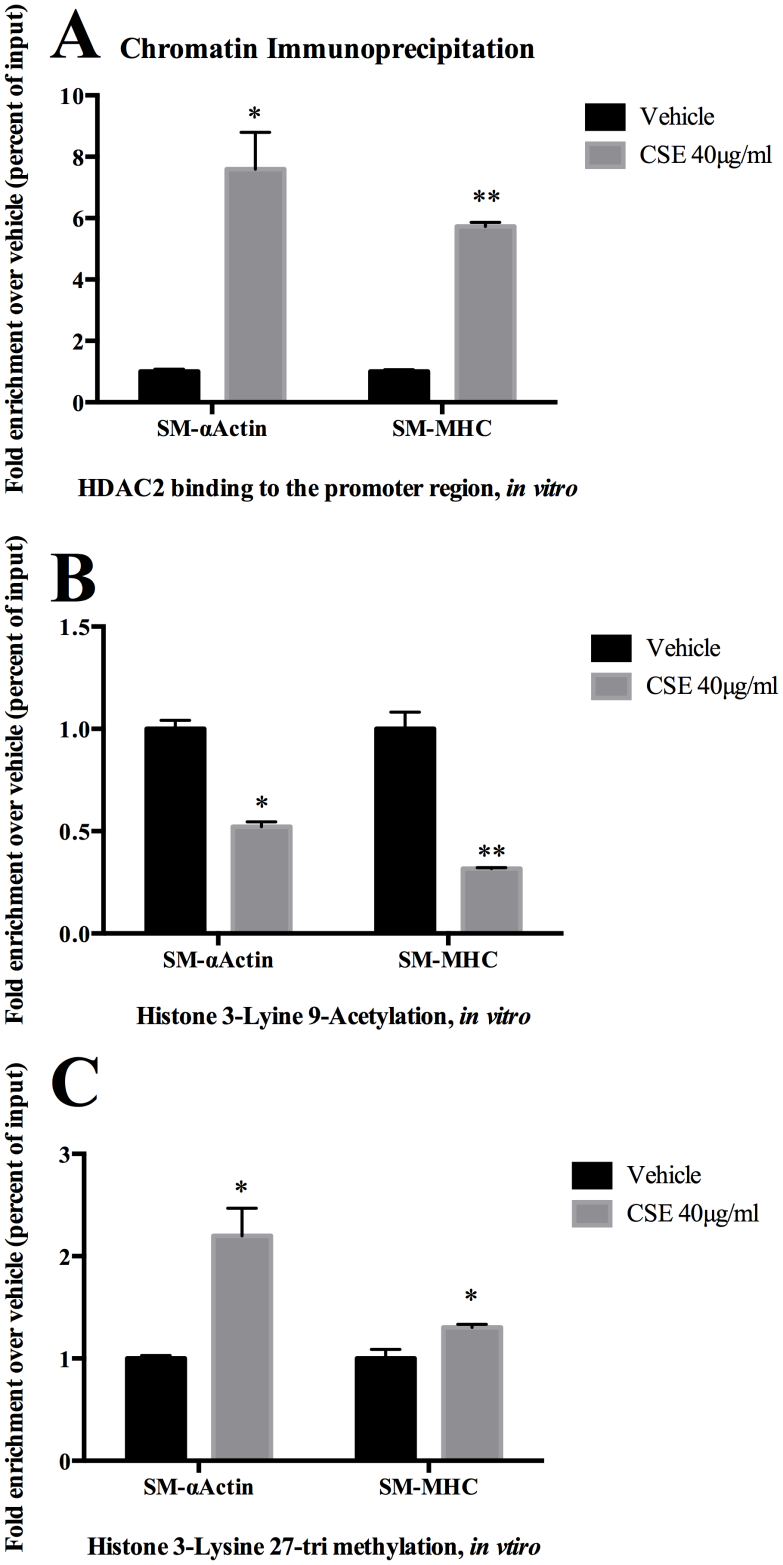
CSE-induced suppression of SMC marker genes was accompanied by recruitment of HDAC2, promoter hypoacetylation, and changes in promoter methylation. A) Cultured cerebral vascular SMC were treated with CSE (40 µg/ml) for 2 hours. Association of HDAC2 to the CArG containing promoter region of SMC marker genes (SM-α-actin and SM-MHC) was determined with ChIP assay using anti-HDAC2 antibody. Values represent fold-increase over vehicle. B & C). Similar to above, ChIP assays were performed with the following antibodies: anti-H3K9Ac, and anti-H3K27triMe. Values represent fold-increase over.

To determine if CSE results in epigenetic alterations that alter vascular SMC marker gene expression, we assessed the effects of CSE exposure on histone modifications. Cerebral vascular SMC exposure to CSE resulted in recruitment of histone deactylase (HDAC) to the promoter region of SM-α-actin and SM-MHC (Figure 9A) and corresponding hypoacetylation of histones at the promoter regions of these genes (Figure 9B). CSE exposure also induced H3K27 trimethylation of histones, which is characteristic of transcriptional suppression [39], [40] (Figure 9C). In rat carotid arteries *in vivo* assays demonstrated a nonsignificant trend towards similar changes in hypoacetylation (data not shown). Taken collectively, these results demonstrate that CSE recruits HDAC2 to the promoter region of SMC marker genes producing hypoacetylation and also results in promoter methylation indicative of gene transcriptional repression.

## Discussion

Smoking is a major cause of vascular associated morbidity and mortality. Despite significant dysregulation in endothelial cells exposed to cigarette smoke, the precise pathological mechanisms remain unclear; specifically, there have been limited studies of SMC. [12], [14], [41], [42] There has been significant progress in understanding SMC biology, but mechanisms underlying control of SMC differentiation in disease states remain unclear. Little is known about vascular SMC phenotypic modulation in the cerebral circulation although there are clear differences in control of vascular SMC differentiation in various vascular beds. [24] Although evidence demonstrates that SMC phenotypic modulation plays a role in the pathogenesis of vascular injury and atherosclerosis, [8], [27], [28], [29] it has not been assessed following exposure to cigarette smoke. In this report, we provide novel evidence demonstrating that CSE regulates cerebral vascular phenotypic modulation through downregulation of SMC differentiation genes and upregulation of pro-inflammatory/matrix remodeling genes via a KLF4/myocardin dependent pathway. Similar alterations were found early during an *in vivo* model of carotid disease providing important implications for the mechanisms by which cigarette smoke may contribute to atherosclerosis during ischemic stroke, intracerebral hemorrhage, and cerebral aneurysm formation.

Studies in cultured peripheral SMC have shown that these cells retain significant plasticity. [23], [24] In response to a number of stimuli, aortic cultured SMC can change into cells primarily concerned [27] with contraction or regress to a pro-inflammatory, pro-matrix remodeling phenotype. [23], [25], [26], [27], [28], [29], [30] In healthy arteries, SMC assume a contractile role in regulation of blood flow and pressure. Studies have found that myocardin is a key mediator of phenotypic modulation [23], [25], [26], [27] promoting SMC differentiation through upregulation of SMC marker genes including SM-MHC, SM-α-actin and SM-22α. [26], [29], [43] Unlike SM-α-actin whose main role is in contraction [44], there are a number of SMC genes primarily involved in cellular structure and integrity; for example, beta-actin which has not been found to be involved in SMC phenotypic modulation in response to a number of stimuli. [23], [24], [45] Following vascular injury myocardin is inhibited by KLF4 and SMC induced inflammatory mediators. [25], [26], [27] Prior studies of cerebrovascular plasticity have provided limited assessment of vascular SMC phenotypic modulation. Findings from the present study demonstrate that vascular SMC phenotypic modulation occurs at least in part via similar mechanisms within the cerebral circulation as compared to the peripheral circulation.

Cigarette smokers are known to have global upregulation of inflammatory markers and immune cells. [20], [21], [22] This likely leads to increased inflammatory cells and cytokines in cerebral endothelial cells as demonstrated in cell cultures and animal studies following exposure to cigarette smoke. [14], [15], [16], [17], [18], [19] Observations in the present study demonstrate that CSE induces significant phenotypic modulation of cerebral vascular SMC characterized by shift from a contractile phenotype to a pro-inflammatory, pro-matrix remodeling phenotype. We have found both *in vitro* and *in vivo* that CSE mediates downregulation of myocardin, SM-α-actin, SM-MHC and SM-22-α and upregulation of MCP-1, MMPs, TNF-α, IL-1β, NF-κB in a dose-dependent manner. This supports prior studies that have demonstrated alterations in proliferation in vascular SMC [46], [47], [48] as well as upregulation of inflammatory modulators following exposure to cigarette smoke [49].

These molecular changes have been shown to be key steps in the initiation of atherosclerosis and vascular disease. MCP-1 attracts monocytes and migration into atherosclerotic lesions. [50] MMPs are important in chemokine/cytokine activation, play a key role in cell proliferation, migration, and adhesion, and have been shown to degrade key structural proteins. [51] TNF-α plays a pivotal regulatory role in the inflammatory cascade through recruitment of immune cells, initiation of smooth muscle cell apoptosis, and atherosclerotic plaque destabilization following extracellular matrix remodeling. [52], [53] We have previously found that TNF-α may promote an inflammatory SMC phenotype. [54] IL-1β also plays an important role in recruitment of inflammatory cells into atherosclerotic lesions. [55], [56] Additionally, in aortic cultured SMCs, IL-1β promotes SMC phenotypic modulation through increased expression of pro-inflammatory genes and NF-κB helps promote inflammatory gene expression in response to IL-1β. [57] Although the role of key inflammatory cytokines and immune cells have been recognized in cigarette-induced vascular injury and play a primary role in the initiation of atherosclerosis, [15], [16], [17], [19] the role of cerebral vascular SMC phenotypic modulation has been largely ignored.

Vascular SMC phenotypic modulation has shown to play a key role in peripheral vascular atherosclerosis, [38] a key element of carotid and cerebrovascular disease. [58], [59] Additionally, smoking is a significant modifiable risk factor for carotid and cerebrovascular diseases including ischemic stroke, intracerebral hemorrhage, and cerebral aneurysm formation. [7], [9], [10], [11], [30] Results above demonstrate that CSE can directly result in marked downregulation of SMC contractile marker genes and upregulation of pro-inflammatory/matrix remodeling genes. Prior studies have found that MCP-1, TNF-α, IL-1β, NF-κB, and MMP-9 are either requisites or significant mediators of stroke [58], [59] and cerebral aneurysm formation. [9] Additionally, KLF4 has been shown to activate macrophages, [60] key inflammatory cells involved in carotid and cerebrovascular disease. [9], [58], [59] Along with TNF-α and other key inflammatory mediators, this may regulate apoptosis and phagocytosis of vascular SMC in atherosclerotic plaques and may lead to plaque progression and rupture. [38], [58], [59] Thus, vascular SMC phenotypic modulation following exposure to cigarette smoke is likely a key element in the pathogenesis of carotid and cerebrovascular disease through recruitment of vascular adhesion molecules and inflammatory cells. Following exposure to cigarette smoke, vascular SMC change from a contractile phenotype to one characterized by pro-inflammatory/matrix remodeling, which may eventually lead to apoptosis with loss of both phenotypes and atherosclerotic plaque rupture (Figure 3).

Although a number of studies have found that exposure to cigarette smoke results in upregulation of key inflammatory mediators, [12], [13], [14], [15], [16], [17], [18], [19], [20], [21], [22] and these alterations in inflammatory mediators are integral elements behind vascular injury, [30] the mechanism behind this regulation has not been elucidated. KLF4 is a pluripotency factor involved in reprogramming of somatic cells. [32] Prior studies have also shown that KLF4 was required for phenotypic modulation following vascular injury [28] and experimental atherosclerosis. [29] The SMC MHC gene [26] and myocardin promoter [25] also hold KLF4 binding sites and are inhibited during SMC phenotypic switching to an inflammatory phenotype. Results of the present *in vitro* and *in vivo* experiments demonstrate that CSE-induced suppression of SMC genes was dependent on the transcription factor KLF4. Additionally, epigenetic control mechanisms including alterations in histone modifications characteristic of transcriptional suppression [39], [40], [61] provided further evidence that KLF4 regulates cerebral vascular SMC phenotypic modulation through inhibition of myocardin-mediated activation of SMC genes. [28], [62].

Although KLF4 plays an integral role in suppression of SMC genes, additional elements may be required as studies have found additional repressor pathways in SMC phenotypic modulation. [63] Further studies that assess cigarette exposure during *in vitro* and *in vivo* disease states would be beneficial in defining SMC phenotypic modulation alterations in cerebrovascular atherosclerosis as well as clinically relevant entities such as stroke and cerebral aneurysm rupture. Although vascular SMC phenotypic modulation following exposure to cigarette smoke is likely a key element behind the pathogenesis of vascular disease, there is likely a multifactorial role that includes inflammation, hemodynamic stress, and genetic alterations.

In conclusion, this is the first study to assess pathological vascular SMC phenotypic modulation induced via cigarette smoke in the cerebral circulation. It provides novel *in vitro* and *in vivo* evidence that CSE profoundly suppresses expression of cerebral SMC differentiation genes and promotes matrix remodeling/inflammatory gene expression. These processes are, at least in part, regulated by CSE induced upregulation of KLF4 and myocardin inhibition. This provides a mechanism whereby cigarette smoke may induce vascular SMC phenotypic modulation and provides an important pathway for cigarette smoke to contribute to carotid and cerebrovascular atherosclerosis during ischemic stroke, intracerebral hemorrhage, and cerebral aneurysm formation. Understanding such fundamental pathological mechanisms may ultimately translate to the design of effective therapies for these important diseases that are a leading cause of death and disability in the United States.
